# A Hero for Our Time

**DOI:** 10.1093/biosci/biac114

**Published:** 2023-03-17

**Authors:** Charles Hall

**Affiliations:** State University of New York's College of Environmental Science and Forestry, Syracuse, New York, United States

**Herman Daly's Economics for a Full World: His Life and Ideas.** Victor, Peter A. Routledge, 2022. 320 pp., illus. ${\$}$44.95 (ISBN: 978–0,367,556,952).

Biologists are increasingly called on to help make economic (or, more precisely, monetary) evaluations as political decisions are turned over to the market. This includes ecologists, who interact with economists while evaluating ecosystem services; field assayers helping to quantify cost–benefit analyses; biologists assessing the loss of organs or functions; and the like. Most biologists probably just accept that conventional economics is a legitimate science and go about their business as a biologist. Although there is no reason not to make these monetary estimates (because the public is likely to understand them better than the more esoteric criteria we might choose instead), it is also important for biologists to understand that conventional, contemporary (i.e., neoclassical) economics has many serious problems, to the degree that many believe the entire process to be quite suspect or even fraudulent. It is not just that economics is criticized as a tool used routinely to justify inequitable distributions and the excessive exploitation of nature (e.g., Piketty 2014). Much more fundamentally, conventional economics violates criteria we routinely require in the natural sciences. These include operating within the laws of thermodynamics, the use of appropriate boundaries, and using the scientific method to derive basic methodology (Leontief 1983, Hall et al. 2001, Erickson 2022, Victor 2022). At a minimum, biologists should know more about what they are doing when they are contributing to economic analyses.

Peter A. Victor's latest book is one of the best places to start understanding what is wrong with the uncritical use of conventional economics. Herman Daly and his PhD adviser, Nicholas Georgescu Roegen, understood the fundamental discrepancies between conventional economics and the natural sciences better than nearly any other investigator. Georgescu Roegen, not always an easy person to deal with, went to his grave bitter that he did not win a Nobel prize (which, in my opinion, he well deserved) for showing clearly that economic production was basically about energy, a factor essentially ignored by most economists. Victor shows how Daly extended that view in many ways, including the ultimate limits of energy on economic growth. The basic question is how one should consider the role of energy: Economists view energy as simply another commodity—and not even a very important one, since energy is only some 5% or 10% of the monetary value of gross domestic product. But according to Daly, the economists have it exactly backward. Energy is important *because* it is cheap. A barrel of oil, which you can buy for ${\$}$100 or so, can do the physical work of a strong laborer over 2 years—which would cost you some ${\$}$80,000. For some peculiar reason, contemporary (neoclassical) economists include only capital and labor in their production functions. When they do that, they can explain only about half of economic growth over decades. The rest, called by economists the *Solow residual*, cannot be explained in that model, so it is attributed to “technology.” But when Reiner Kummel included energy (which, as Daly says, is essential) the Solow residual disappears, and all economic growth is explained.

**Figure 1:**
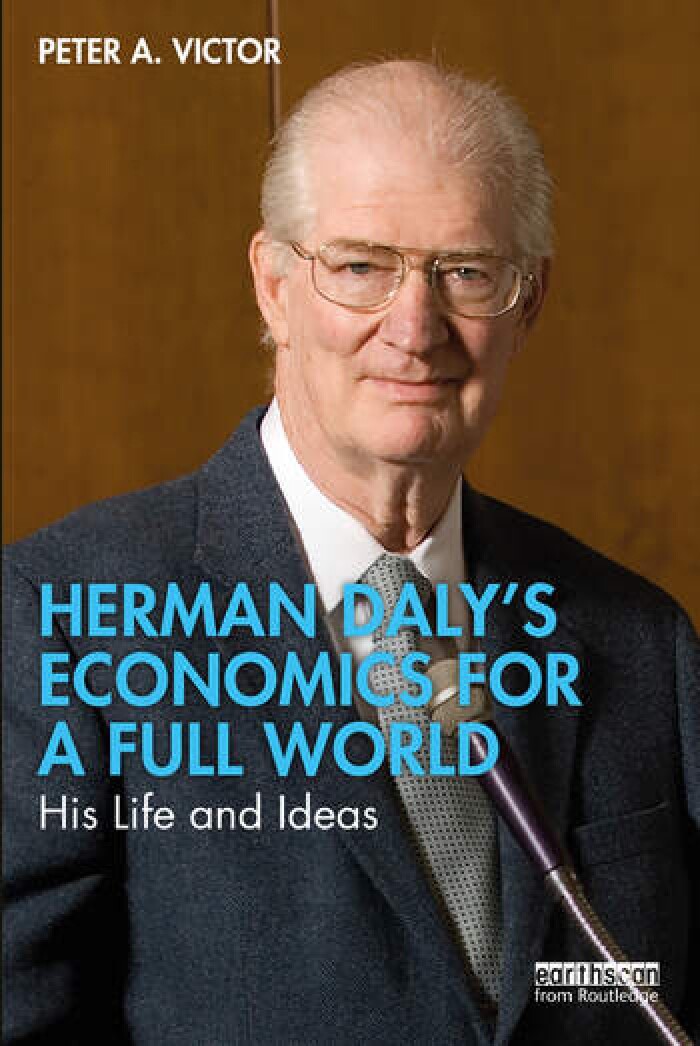


Peter Victor is, like me, very influenced by Herman Daly's thinking, especially his earlier thinking. Although Herman was very much an economist, he thought in a clear, basic way that most natural scientists will find familiar. For example, he says that economists see nature as a subset of the economy, one that can be priced and entered into larger economic calculations of value. The reality, however, is the converse, that our economy is a subset of nature and must live and operate within nature's boundaries, limits, and possibilities. As we increasingly destroy species and ecosystems for financial gain, the shortsightedness of this reversal is increasingly obvious and must be compensated for with ever growing expenditures of fossil energy.

## Growth in a Full World

But Herman Daly was even more interested in *why* we were doing economics and economic analysis and what else we leave out. He argued that economists constantly confused means and ends, most importantly where economic growth, a means to the ends of human welfare, became and end in itself. Given the enormous destruction of nature and many human societies from the pursuit of economic growth, it was obvious to Daly that we needed to cease economic growth and focus on the real problems of improving human welfare. An important part of this would be replacing more materialistic goals with more humane and spiritual ones, the latter especially important to Herman.

Conventional economics requires growth to meet its increasing need for material progress and return to capital investment. Such growth allows society to sidestep dealing with obvious issues such as distribution and even pollution. Herman Daly is most widely known for an antidote to that, the concept of steady-state economics, an approach to economics that does not rely on growth to be successful. Victor finds this approach essential as we transition from an “empty” world to a “full” world. Daly stated simply what he found to be an obvious truth, one that is very familiar to biologists. A carrion beetle and its descendants may have a good run of it while a large carcass exists, but biologists know that, eventually, the resource will be gone, and the beetles will crash—unless another carcass comes their way. For humans, fossil fuels are such a carcass, and they have allowed exponential growth of humans and their economies until about now. For many, the end of this growth is clearly in sight. Biologists know full well that nature has its limits—that organisms or their populations cannot grow indefinitely, that the availability of energy and other resources limits what kinds and levels of ecosystems we have and what the options for growth might be.

Victor chronicles how most conventional economists do not see it that way, believing that, somehow, economic growth, which has been strong for 200 years, can continue indefinitely. Even so, many European economists have been commenting on what they call “secular stagnation” and are baffled by it. Why have European economies ceased to grow? The answer is obvious to anyone who follows Daly and his ilk: The North Sea oil reached its peak two decades ago, after giving the British economy a big kick in the pants (that Margaret Thatcher liked to take credit for despite her limited role). Now, Europe has to import two-thirds of its energy while trying to maintain a lifestyle once lived only by kings and queens. In addition, all around the world, the easy resources, both biological and mineral, are gone, requiring more energy to get the next unit. Humans see the results as inflation and tend to blame their leaders for something that is the inevitable result of growth and the depletion it causes.

The book is clearly written, is well organized, is scholarly, and possesses a strong bibliography. Victor makes frequent use of quotations from Daly himself, and, in this sense, the book is an excellent introduction to Daly's main ideas in his own words. The book says remarkably little about the International Society of Ecological Economics (and its journal), which Daly helped found but which many find (I think, including Daly) has failed to live up to its promise by succumbing to too much pricing of the unpriceable and not enough criticism of basic economics (e.g., Ropke 1984, Melgar-Melgar and Hall 2019).

My only particular criticism is that Victor should be more furious that Daly's good common sense and clear explanations have had so little impact. Perhaps it is because Herman Daly was a real Southern gentleman, gentle in his demeanor and even gentle when defending himself against the many unwarranted attacks he sustained.

We live in a world of illusions and lies, propagated by fake news broadcasts, politicians, economists, and sometimes scientists. Of particular concern to me (and Victor) is the series of fairy tales that passes for neoclassical economics. Although it is often cloaked in seemingly sophisticated and complex, impenetrable mathematics, Daly, Victor, and I find that conventional economics does not pass muster with real natural scientists. My heroes are those who cut through the perceived wisdom to show the actual essence of issues rather than the arcane mathematical complexities. Chief among them is Herman Daly. If you are not familiar with Herman's critique of economics, I strongly recommend you read this book, especially if you undertake economic work. For those of us who work daily with Herman's concepts, they are laid out here so logically and naturally that it is more of a good review than anything remarkably new. There are other alternatives to conventional economics available, including the fields of ecological economics and my favorite, biophysical economics (e.g., Hall and Klitgaard 2017). In my opinion, these should be given as a first year of economics, to provide young people a chance to choose. But like Daly, I am not optimistic. The “science” of economics is simply too entrenched, and the critics have too little influence with those who benefit from the logical and distributional fallacies of our conventional—and often ­foundering—­economic system.

How to Contact AIBS BioScience

Advertising, print and online: *jnlsadvertising@oup.com*
Classified advertising: *info@kerhgroup.com 855–895-5374*
Online: *https://academic.oup.com/bioscience*
Permissions: *journals.permissions@oup.com*
Submission inquiries: *bioscience@aibs.org* 703–674-2500 x. 326
Subscriptions: Individual *membership@aibs.org 703–674-2500* x. 247

AIBS

Membership Records: *membership@aibs.org 703–674-2500* x. 247
Community Programs: *dbosnjak@aibs.org 703–674-2500* x. 247
Public Policy Office: *jpandey@aibs.org 202–628-1500* x. 250
Scientific Peer-Review Services: *sglisson@aibs.org 703–674-2500* x. 202
Web/IT Services: *jwagener@aibs.org 703–674-2500* x. 107

## Author Biographical

Charles Hall is Professor Emeritus at the State University of New York's College of Environmental Science and Forestry, in Syracuse, New York, in the United States.

## References

[bib1] Erickson JD . 2022. The Progress Illusion: Reclaiming Our Future from the Fairytale of Economics. Island Press.

[bib2] Hall CAS , Klitgaard K. 2017. Energy and the Wealth of Nations: An Introduction to BioPhysical Economics. Springer.

[bib3] Hall CAS , Lindenberger D, Kümmel R, Kroeger T, Eichhorn W. 2001. The need to reintegrate the natural sciences with economics. BioScience 51: 663–673.

[bib4] Leontief W . 1982. Academic economics. Science 217: 104–107. 17770240 10.1126/science.217.4555.104

[bib5] Melgar-Melgar RE , Hall CAS. 2019. Why ecological economics needs to return to its roots: the biophysical foundation of socio-economic systems. Ecological Economics 169: 106567. 10.1016/j.ecolecon.2019.106567.

[bib6] Piketty T . 2014. Capital in the Twenty-First Century. Arthur Goldhammer, trans. Belknap Press. 10.1111/1468-4446.12115 25516350

[bib7] Ropke I . 1984. The early history of modern ecological economics. Ecological Economics 50: 293–314.

[bib8] Victor PA . 2022. Herman Daly's Economics for a Full World. Routledge.

